# Investigating the Time-Varying Nature of Medication Adherence Predictors: An Experimental Approach Using Andersen’s Behavioral Model of Health Services Use

**DOI:** 10.3390/pharmacy13020053

**Published:** 2025-04-09

**Authors:** Vasco M. Pontinha, Julie A. Patterson, Dave L. Dixon, Norman V. Carroll, D’Arcy Mays, Karen B. Farris, David A. Holdford

**Affiliations:** 1 Department of Pharmacotherapy and Outcomes Science, VCU School of Pharmacy, Richmond, VA 23298, USA; 2 Center for Pharmacy Practice Innovation, VCU School of Pharmacy, Richmond, VA 23298, USA; 3 Department of Statistical Sciences and Operations Research, VCU College of Humanities & Sciences, Richmond, VA 23220, USA; 4 College of Pharmacy, University of Michigan College of Pharmacy, Ann Arbor, MI 48109, USA

**Keywords:** medication adherence [MeSH], pharmacoepidemiology [MeSH], aging, multitrajectory group-based models, group-based trajectory model, risk factors

## Abstract

Medication adherence is a crucial factor for managing chronic conditions, especially in aging adults. Previous studies have identified predictors of medication adherence. However, current methods fail to capture the time-varying nature of how risk factors can influence adherence behavior. This objective of this study was to implement multitrajectory group-based models to compare a time-varying to a time-fixed approach to identifying non-adherence risk factors. The study population comprised 11,068 Medicare beneficiaries aged 65 and older taking select medications for hypertension, high blood cholesterol, and oral diabetes medications, between 2008 and 2016. Time-fixed predictors (e.g., sex, education) were examined using generalized multinomial logistic regression, while time-varying predictors were explored through multitrajectory group-based modeling. Several predisposing, enabling, and need characteristics were identified as risk factors for following at least one non-adherence trajectory. Time-varying predictors displayed an alternative representation of those risk factors, especially depression symptoms. This study highlights the dynamic nature of medication adherence predictors and the utility of multitrajectory modeling. Findings suggest that targeted interventions can be developed by addressing the key time-varying factors affecting adherence.

## 1. Introduction

Non-adherence to medications is a major barrier to achieve desired outcomes, improve clinical outcomes, and improve health status [1,2,3,4,5,6,7,8]. It is estimated that only 50% of patients with chronic conditions are adherent to their treatment plan, which results in significant clinical and economic burdens. Every year in the United States, non-adherence to medications results in 125,000 deaths and health care costs up to USD 289 billion [9,10,11,12]. Given the tremendous impact in outcomes, the Centers for Medicare and Medicaid Services (CMS) administers the Star Ratings program, which rewards health plans based on their performance in health plan metrics, including medication adherence. The drugs considered in this quality metric program include renin–angiotensin system antagonists for hypertension, statins for hypercholesterolemia, and diabetes drugs excluding insulin (i.e., biguanides, sulfonylureas, thiazolidinediones, dipeptidyl peptidase-4 inhibitors, incretin mimetics, meglitinides, or sodium-glucose cotransporter 2 inhibitors).

In 2003, the World Health Organization issued a report highlighting the multifactorial causes of non-adherence, including socioeconomic, health care team and health system, disease-related, therapy-related, and patient-related factors [8,13]. These factors align with Andersen’s Behavior Model of Health Services Use (ABM), a widely used theoretical framework in health service research. Originally developed to study the family health service use, ABM is now used to explain interactions with medication use [14,15]. Its dimensions—predisposing characteristics (e.g., socio-demographic, social structure, and health beliefs), resources (personal, family, and community), and need (health status, comorbidities, treatment complexity, and patient’s independence)—overlap conceptually with the WHO’s non-adherence factors. Despite the seemingly broad agreement to using this ABM as a theoretical framework for studying health service use, the operationalization of specific items included in predisposing characteristics, enabling factors, and need resources has been inconsistent in previous studies [16].

**Figure 1:**
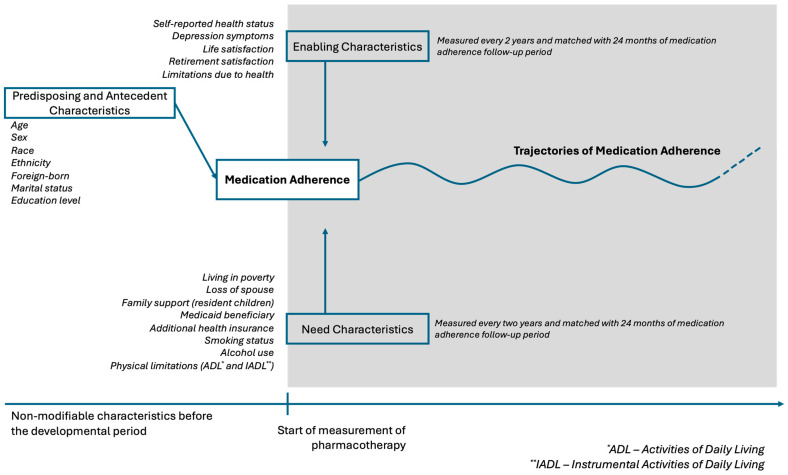
Study conceptual framework.

In order to determine patterns of medication adherence behavior and predictors of poor performance, researchers increasingly use group-based trajectory modeling (GBTM) to analyze patterns of medication adherence across various prescription drugs [17,18,19,20,21,22,23]. Unlike categorizing patients as adherent or non-adherent, GBTM identifies similar adherence trajectories over time [24]. Previous studies primarily focused on predisposing characteristics, such as education, sex, ethnicity and race, or a single need characteristic, like comorbidities. However, adherence trajectories and their predictors can change over time, as risk factors for non-adherence do not occur in isolation or simultaneously. Traditional methods identify predictors by estimating their effects while holding other variables constant, focusing on fixed aspects influencing behavior over time [25,26]. Yet, time-varying factors like income, Medicaid eligibility, family support (e.g., spouse loss, household changes), and ambulatory independence may have a dynamic combined effect on medication adherence. Multitrajectory group-based modeling, an extension of GBTM, examines how such dynamic factors contemporaneously influence outcomes. This conceptual study used multitrajectory group-based models to describe the time-varying predictors of medication adherence trajectories following the ABM theoretical framework. This research addresses a fundamental gap in the medication adherence literature concerning the dynamic effect of risk factors of non-adherence (Figure 1).

## 2. Methods

### 2.1. Group-Based Trajectory Models (GBTMs) of Medication Adherence

This study ran a post hoc analysis of GBTM models previously estimated from monthly measurements of the proportion of days covered (PDC) to describe the longitudinal patterns of medication adherence of Medicare beneficiaries ≥65 years old between January 2008 and December 2016 [27]. The GBTM models were derived from participants from the Health and Retirement Study (HRS), which is a longitudinal panel study with a nationally representative sample of approximately 20,000 people in the United States sponsored by the National Institute of Aging (grant number NIA U01AG009740) and is conducted by the University of Michigan. Patients were taking select antihypertensives, including renin–angiotensin–aldosterone system inhibitors (RAASs), HMG Co-A reductase inhibitors (statins), or oral diabetes medications during the follow-up period [27]. The inclusion and exclusion criteria are described elsewhere, as well as a complete list of drugs included in the GBTM models [27]. GBTM models were estimated for antihypertensive drugs, statins, and diabetes medications (excluding insulin). Briefly, medication adherence was quantified using monthly measurements of PDC at the drug class level. This approach ensured that adherence measures were not influenced by drug within-class or impacts from brand-to-generic switches. In short, the GBTM models yielded three different models based on the drug class: select antihypertensives, with 3 trajectories (high-to-very high adherence, slow decline, and rapid decline); statins, yielding 5 trajectories (high-to-very high adherence, slow decline, low then increasing adherence, moderate decline, and rapid decline); and oral antidiabetics, which revealed 6 trajectories (high-to-very high adherence, slow decline, high then increasing adherence, low then increasing, moderate decline, and rapid decline). This study was approved by the Virginia Commonwealth University Internal Review Board (IRB) and the University of Michigan. HRS-linked administrative health claims data files from Medicare were obtained through CMS’s 3rd party data provider Medicare Research Information Center (MedRIC).

### 2.2. Predictors of Medication Adherence Trajectories

The conceptual framework of this study was based on ABM. However, it was important to ensure consistency with previous research. Therefore, the operationalization of variables for each of the dimensions of the ABM theoretical framework followed those as prescribed in Babitsch and colleagues (Table 1) [16].

Measurements informing the covariates were obtained from the RAND longitudinal data file of the HRS public survey, including Sections A through K, respective to the period of analysis of medication adherence: 2008–2016 [28]. The relationship between predisposing, antecedents, enabling, and need characteristics (Appendix A) was examined in two ways: Firstly, using a time-stable approach, in which the last observation of each characteristic was investigated in the appropriate risk factor variation regression model. Secondly, through a time-varying approach in which repeated measures of each characteristic were explored in a multitrajectory group-based method. To minimize the impact of missing data, the last observation was carried forward in the time-varying approach. Only complete cases were considered in the final analysis. All statistical analyses were conducted using STATA MP 17 [29].

### 2.3. Time-Stable Predictors

A risk factor variation was implemented in the GBTM medication adherence model to examine which non-modifiable covariate was associated with membership to medication adherence trajectories for each of the 3 pharmacotherapeutic drug classes. This was achieved by performing a generalized logistic regression to each of the group-based trajectory models, in which time-stable covariates were tested for their ability to change group-membership probability [30]. A generalized logistic regression is an optimal approach because the parameters for each trajectory $\theta_{j}$ of the multinomial logistic regression are able to denote the probability (*P*) of an individual *i*’s membership in group ($\pi_{j}{(x}_{i})$), given the vector of variables that determine trajectory group membership (*x_i_*) (Equation (1)) [25,31,32]. Effectively, the effect of each vector of non-modifiable risk factor over time is modeled without loss of generality, where *θ*_1_ = 0 (Equation (2)) [30].

(1) $$P\left(Y_{i}\right)=\sum_{j}^{i}\left[\frac{e^{x_{i}\theta_{j}}}{\sum_{j}e^{x_{i}\theta_{j}}}\right]P^{j}\left(Y_{i}\right)$$

Equation (1)—Unconditional probability of *i* (individual) in any of the group-based trajectory model.

(2) $$\pi_{j}\left(x_{i}\right)=\frac{e^{x_{i}\theta_{j}}}{\sum_{j}e^{x_{i}\theta_{j}}}$$

Equation (2)—Vector of each time-stable risk factor over time.

Each covariate was investigated individually, followed by an adjusted model including all covariates found to be statistically significant in predicting membership for at least one medication adherence trajectory. Regression estimates, odds ratios, standard errors, and *p*-values were estimated to demonstrate the strength of association between each covariate and trajectory membership. To examine the potential for multicollinearity, variance inflation factor (VIF) was computed to determine by how much each risk factor estimate is increased because of the high correlation with other risk factors. When VIF is equal to 1, the coefficient of determination (R^2^) = 0, which means that the risk factor is not linearly related to other variables [33]. Therefore, a VIF greater than 5 was considered to be an indication of multicollinearity [34].

### 2.4. Time-Varying Predictors

A multitrajectory group-based model was used to examine how time-varying covariates influence membership probabilities across medication adherence trajectories for hypertension, hypercholesterolemia, and diabetes. This model incorporates the previously identified adherence trajectories while simultaneously plotting changes in time-varying predictors [25]. Similar methods have been applied in studying chronic kidney disease [35]. Unlike standard GBTM, this approach calculates conditional probabilities of trajectory membership for additional predictors beyond the first, allowing for a more comprehensive description of multiple risk factors [25,30]. Finally, since measurements of the predictors of medication adherence were obtained every two years, each annual measurement was matched with every 24 months of medication adherence follow-up period.

## 3. Results

In total, 11,068 participants were included in this post hoc analysis as those identified taking RAAS, statins, or oral diabetes medications between 2008 and 2016. The predisposing, enabling, and need characteristics are described in Table 2. Missingness was noteworthy in all the characteristics. The number of observations (n) indicated for each characteristic and the proportion of missingness are represented in Table 2.

### 3.1. Time-Fixed Predictors of Medication Adherence Trajectories

A risk factor variation implemented in each group-based trajectory model of medication adherence is estimated elsewhere [27]. All risk factors included in each trajectory model displayed a VIF < 5, suggesting negligible evidence of multicollinearity (Appendix B). The risk factor variation was achieved by performing a generalized logistic regression with each of the group-based trajectory models, in which time-stable covariates are tested for their ability to change group-membership probability, considering the high-to-very adherence trajectory group as reference. Regression estimates, adjusted odds ratios (aORs), standard errors, and *p*-values were estimated to demonstrate the strength of association between each predisposing, enabling, or need characteristic and the likelihood of medication adherence trajectory membership, assuming the high-to-very high adherence trajectory as the reference group in each model (Table 3, Table 4, Table 5, Table 6 and Table 7).

### 3.2. Time-Varying Predictors of Medication Adherence Trajectories

**Figure 2:**
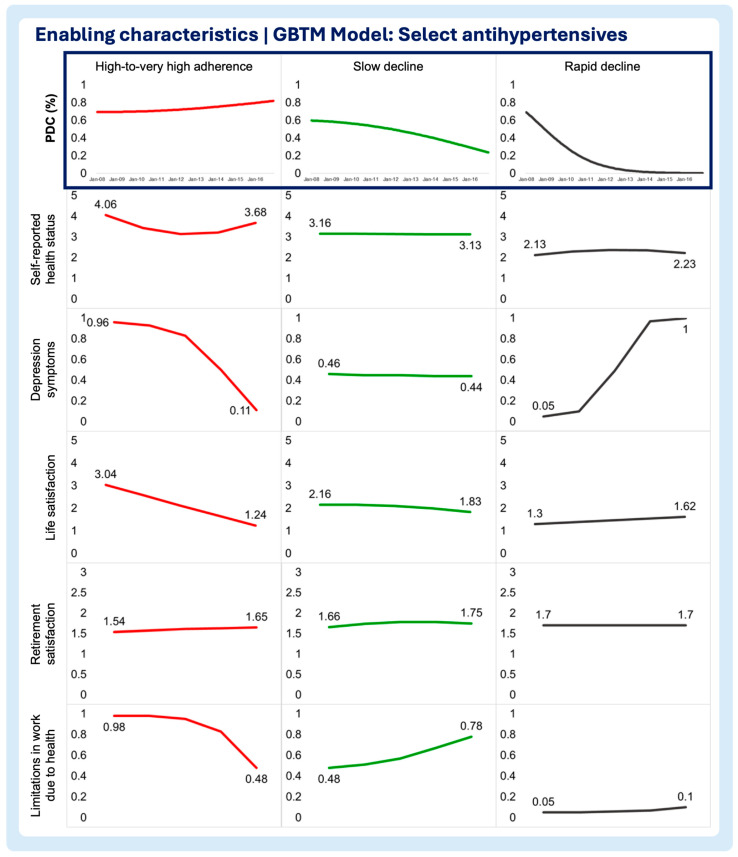
Multitrajectory model of enabling characteristics and select antihypertensive medication adherence trajectory.

**Figure 3:**
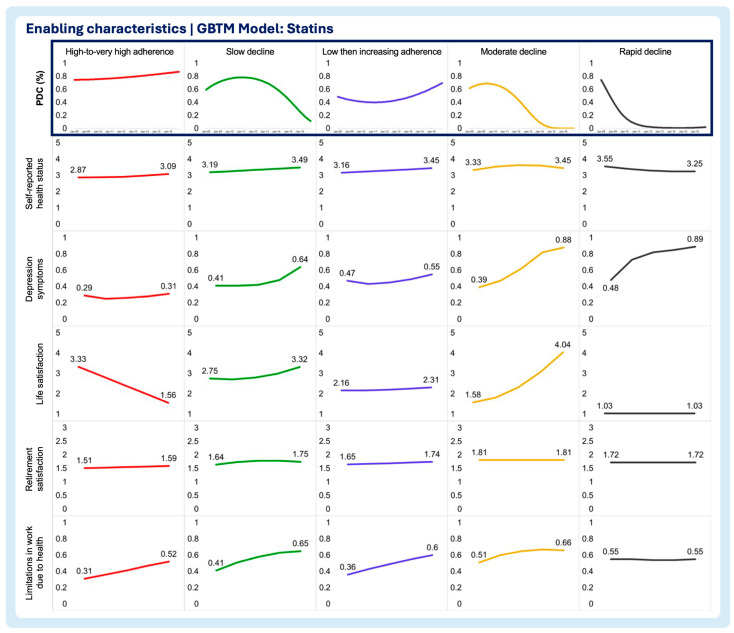
Multitrajectory model of enabling characteristics and statin medication adherence trajectory.

**Figure 4:**
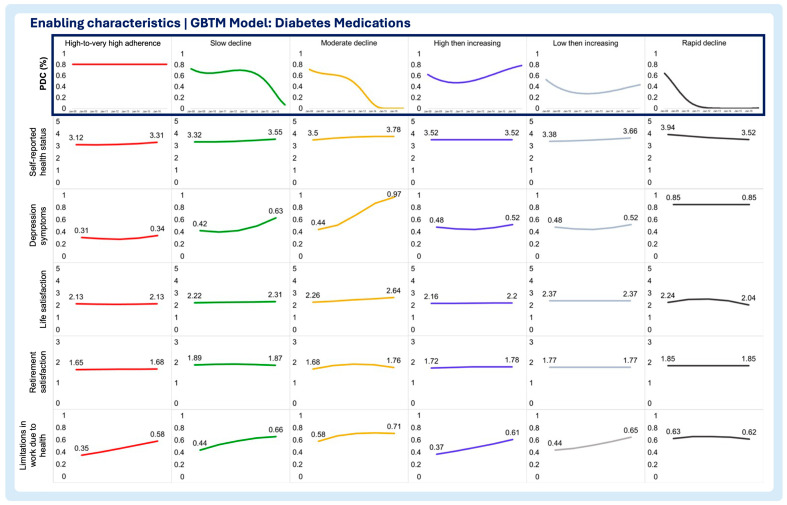
Multitrajectory model of enabling characteristics and diabetes drug medication adherence trajectory.

A multi-group-based trajectory analysis was implemented to investigate if and to what extent each of the time-varying enabling and need characteristics are associated with changes in medication adherence trajectories in each medication adherence model. Figure 2, Figure 3 and Figure 4 describe the multi-group-based trajectory models for the select antihypertensives, statins, and diabetes medications, respectively.

1. Enabling characteristics

- Self-reported health status

**Figure 5:**
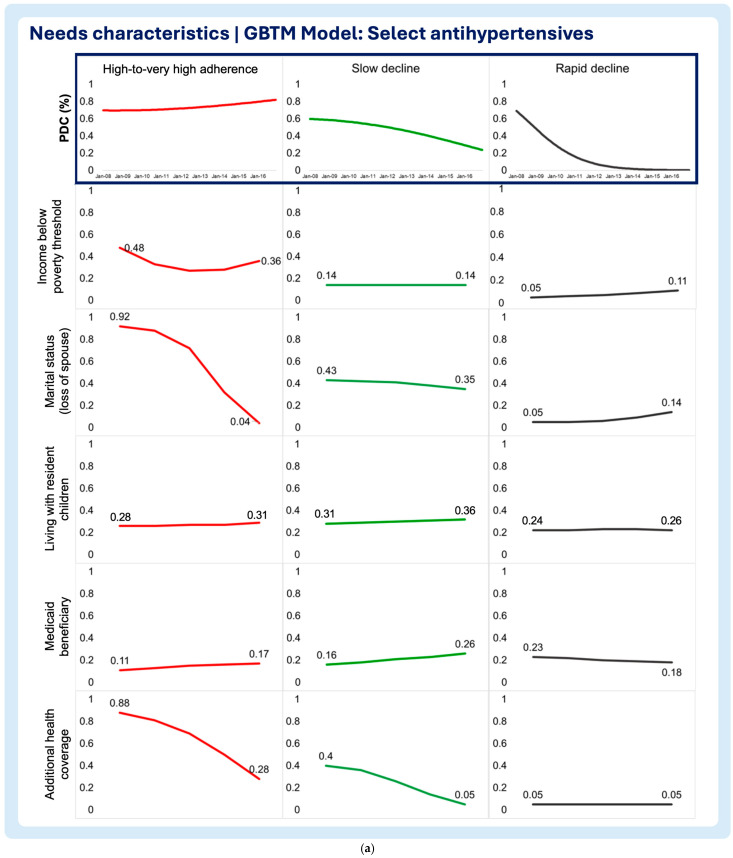

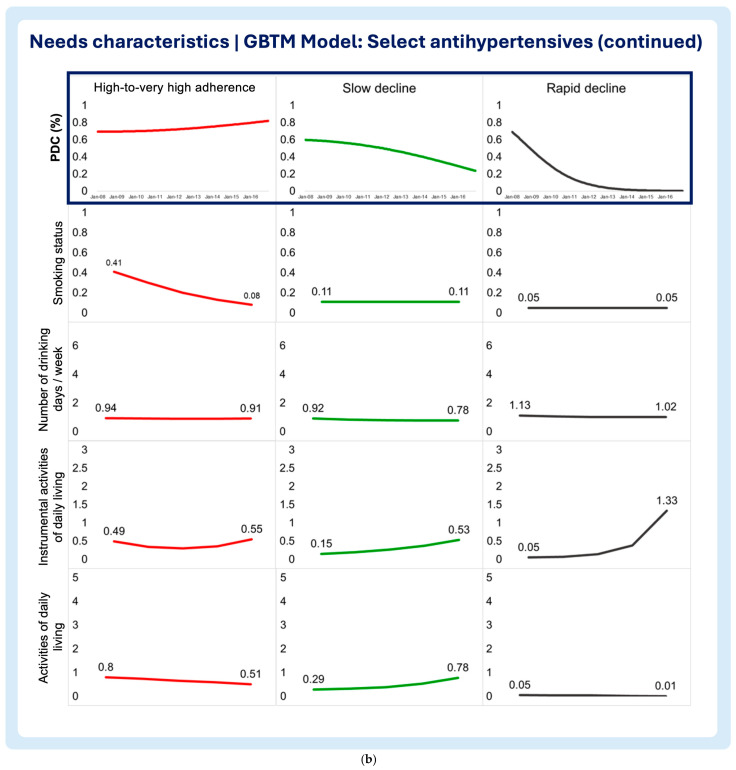
(**a**) Multitrajectory model of need characteristics and select antihypertensive medication adherence trajectory. (**b**) Multitrajectory model of need characteristics and select antihypertensive medication adherence trajectory (continued).

**Figure 6:**
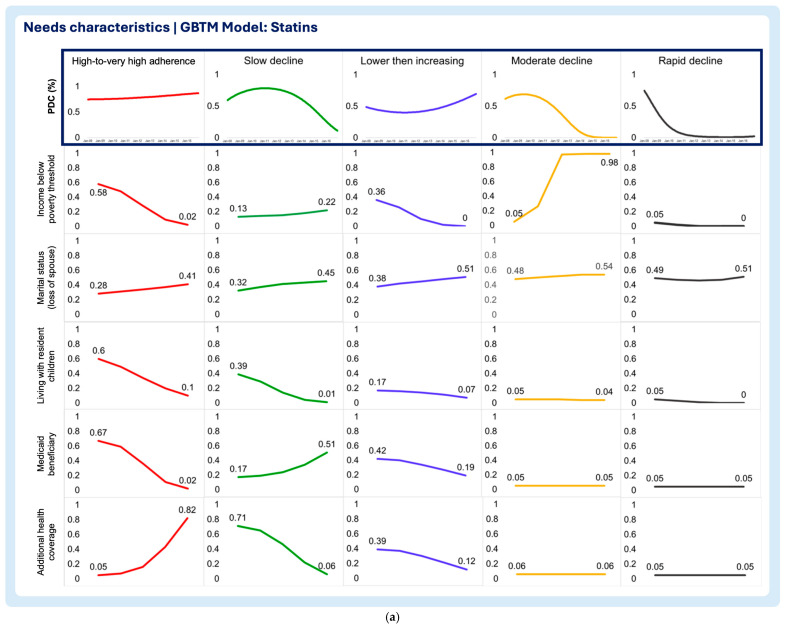

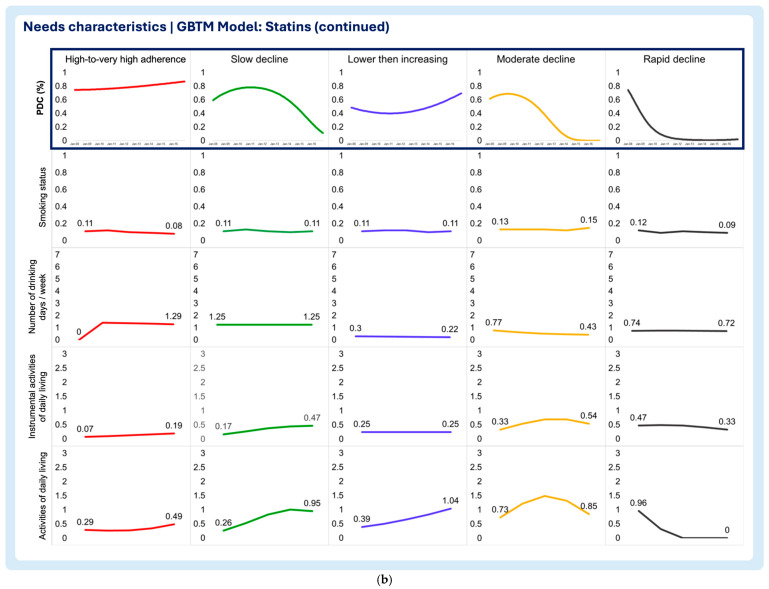
(**a**) Multitrajectory model of need characteristics and statin medication adherence trajectory. (**b**) Multitrajectory model of need characteristics and statin medication adherence trajectory (continued).

**Figure 7:**
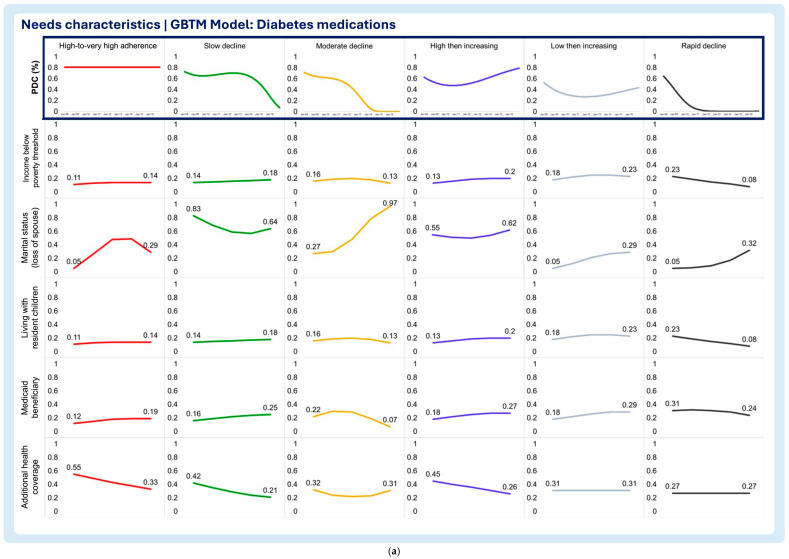

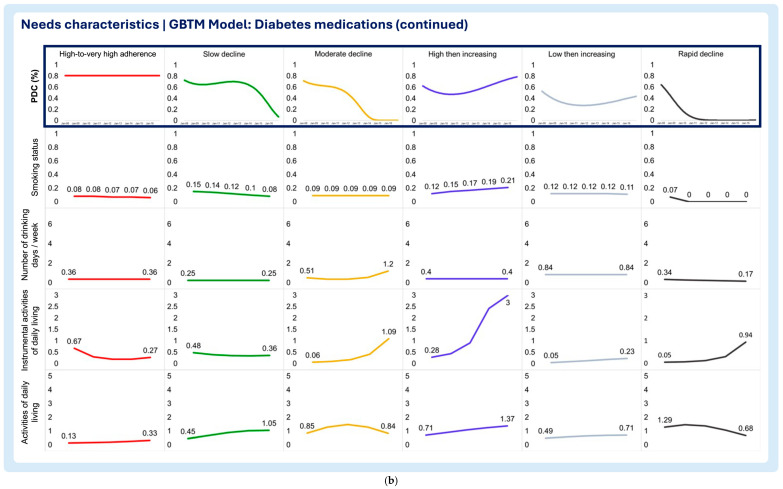
(**a**) Multitrajectory model of need characteristics and diabetes drug medication adherence trajectory. (**b**) Multitrajectory model of need characteristics and diabetes drug medication adherence trajectory (continued).

In the antihypertensives model, better health status correlated with high adherence, with minimal shifts across trajectories. Slow decline showed worse health than high adherence but better than rapid decline (Figure 2, Figure 3, Figure 4, Figure 5, Figure 6 and Figure 7). For statins and diabetes models, health status remained stable across trajectories, ranging from Good to Fair (Figure 3 and Figure 4).

- Depression symptoms

In the antihypertensives model, depression increased in rapid decline, remained stable in slow decline, and declined sharply in high adherence (Figure 2). Statins and diabetes models showed similar patterns, with low, stable depression in high adherence, and sharp increases in moderate and rapid decline trajectories (Figure 3 and Figure 4).

- Life satisfaction

Low adherence groups improved in life satisfaction over time, while slow decline showed stability (Figure 2). Statins showed the sharpest decline in moderate increase trajectories (Figure 3). Diabetes models exhibited no major changes, with all groups scoring as very satisfied (Figure 4).

- Retirement satisfaction

No significant trends emerged across trajectories in any model, with retirement satisfaction remaining constant for every trajectory in all three models (Figure 2, Figure 3, Figure 4, Figure 5, Figure 6 and Figure 7).

- Limitations in work due to health

High-to-very high adherence groups showed declining limitations, while slow decline and other trajectories increased (Figure 2). Trajectories in the statin model saw rising limitations overall, with high adherence starting from the lowest baseline (Figure 3). The diabetes models displayed similar increases across all trajectory groups (Figure 4).

2. Need characteristics

- Household income below poverty threshold

The select antihypertensive model, the high-to-very high adherence group, displayed a clear decline in the probability of living below the poverty threshold, even though other trajectory groups exhibited lower probabilities of living below the poverty threshold throughout the period of analysis (Figure 5a). The high-to-very high and low then increasing trajectories of the statin model display sharp decreases in the likelihood of living below the poverty threshold (Figure 6a). The rapid decline trajectory of the statins exhibited a slight decrease, although the likelihood of living below the poverty threshold was minimal at the beginning of this study (Figure 6a). Additionally, the slow decline trajectory displayed a slight increase in the likelihood of living below the poverty threshold (Figure 6a). In the diabetes medication model, all trajectories exhibit a constant low probability of living below the poverty threshold throughout the follow-up period (Figure 7a).

- Marital status (loss of spouse)

The select antihypertensive model showed that the probability of living without a spouse decreased in the high-to-very high adherence group and for the slow decline group (Figure 5a). Contrastingly, patients in the rapid decline group exhibited the growing probability of being without a spouse (Figure 5a). The statin model showed no differences between trajectory groups, as all reported slight increases in the probability of losing a spouse over time (Figure 6a). In the diabetes medication model, all but the slow decline trajectories display increasing chances of losing a spouse during the follow-up period (Figure 7a). The sharpest increase in the probability of losing a spouse was observed in the moderate decline trajectory (Figure 7a). Notably, the small increase was observed in the high-to-very high adherence (“inverted U” shaped curve) and the low then increasing adherence trajectories (Figure 7a). Even though the slow decline trajectory of the diabetes medication model exhibited a decrease in the likelihood of losing a spouse, the probability of living without a spouse at the baseline and end of the follow-up period was one the highest (Figure 7a).

- Living with resident children

The results show that the probability of living with resident children in the household remained stable throughout the follow-up period with no clear trends or shifts in the select antihypertensive model (Figure 5a). In the statin model, all trajectories exhibited the declining probability of residing with children in the household (Figure 6a). The high-to-very high trajectory in the statin model exhibits the largest probability of living with children at the beginning of this study and also the sharpest decline throughout the follow-up period, followed by the slow decline trajectory group (Figure 7a). Like the select antihypertensives, the diabetes medication model displayed no clear trends with all trajectories displaying a low probability of participants living with their children (Figure 7a).

- Medicaid beneficiary

The probability of being a Medicaid beneficiary was consistently low across all trajectories in the select antihypertensive model, with minimal variation throughout the follow-up (Figure 5a). In the statin model, the high-to-very high adherence trajectory initially had the highest probability, followed by the sharpest decline (Figure 6a). The lower then increasing trajectory showed a smaller decline, while the slow decline trajectory exhibited a notable increase in likelihood (Figure 6a). The moderate and rapid decline trajectories maintained consistently low probabilities (Figure 6a). Similarly, all trajectories in the diabetes medication model displayed consistently low probabilities of Medicaid beneficiary status (Figure 7a).

- Additional health coverage

In the antihypertensive model, the high-to-very high adherence trajectory showed the steepest decline in additional health insurance benefits, with slow decline following a similar but less pronounced pattern (Figure 5a). The rapid decline group remained stable, with minimal benefits (Figure 5a). In the statin model, high adherence showed a notable increase in additional coverage, while slow decline and lower then increasing trajectories decreased (Figure 6a). Moderate and rapid decline groups had consistently low, stable probabilities (Figure 6a). In the diabetes model, additional health insurance benefits declined overall, with high adherence maintaining the highest probability at both baseline and follow-up, while rapid decline showed the lowest (Figure 7a).

- Smoking status

The high-to-very high adherence in the select antihypertensive model displayed the sharpest decline in the likelihood of being a smoker, while the remaining trajectories of this model exhibited sustained a low probability of being smokers (Figure 5b). In the statins, all trajectories displayed a very small and constant probability of being smokers throughout the follow-up period (Figure 6b). The same was observed in the diabetes medication model (Figure 7b).

- Number of drinking days/week

The number of drinking days per week was overall low in all trajectories of the select antihypertensive, statin, and diabetes medication models, with all trajectories exhibiting a constant measure of no more than 1 drinking day per week (Figure 5b, Figure 6b, Figure 7b).

- Instrumental activities of daily living (IADLs)

Difficulty with instrumental activities of daily living seem to generally increase with time, with the rapid decline trajectory exhibiting the sharpest surge in the select antihypertensive model (Figure 5b). Similarly, in the statin model, all but the lower then increasing adherence and rapid decline trajectories exhibit an increase in difficulty with instrumental activities of daily (Figure 6b). The lower then increased trajectory of the statins remained constant throughout the follow-up period, whereas the rapid decline trajectory seems to report slightly less difficulty with instrumental activities of daily during the follow-up period (Figure 6b). Nevertheless, the baseline score of IADLs of the rapid decline in the statin model was the highest compared to all other trajectories in the model (Figure 6b). The diabetes medication model exhibited similar results as the select antihypertensive model, except for the high-to-very high adherence and slow decline trajectories (Figure 7b).

- Activities of daily living (ADLs)

In the select antihypertensive model, the high-to-very high adherence and rapid decline trajectories showed slight decreases in difficulty with activities of daily living (ADLs), while the slow decline trajectory exhibited an increase in tasks requiring assistance (Figure 5b). In the statin model, all trajectories except rapid decline showed increases in ADL difficulty, with the high-to-very high adherence group having the lowest baseline score and smallest increase (Figure 6b). ADL difficulty decreased over time in the statin rapid decline trajectory but started with the highest baseline score (Figure 6b). In the diabetes medication model, ADL scores generally increased, with sharpest rises in the slow decline and high then increasing trajectories (Figure 7b). The rapid decline group, despite improvement, had the highest baseline difficulty at the start of follow-up (Figure 7b).

## 4. Discussion

The purpose of this study was to examine the time-varying nature of risk factors of medication adherence trajectories of aging adults taking chronic medications for hypertension (RASA), hypercholesterolemia (statins), and diabetes (except for insulin). This study applied a multitrajectory group-based model, guided by the ABM framework, to analyze how predisposing, enabling, and need characteristics influence membership in medication adherence trajectory groups. Unlike prior models based solely on administrative claims, this study used HRS survey data to capture enabling and need characteristics. Predictors of medication adherence trajectories were assessed using two approaches: a time-fixed risk model examining the association between predictors and trajectory membership, and a multitrajectory model exploring how adherence trajectories align with changes in time-varying need and enabling characteristics. Importantly, the dynamic trajectory of each risk factor exhibited inconsistent shapes when examined individually for each medication adherence trajectory, while the time-fixed risk factor models exhibited consistency with the predictors of non-adherence to chronic medications [1,2,36,37].

The numerous recent studies examining medication adherence patterns using GBTM is proof that research recognizes that medication adherence is a dynamic behavior that can change with time [27,37,38,39,40,41,42,43,44,45,46,47]. Nevertheless, if one recognizes that medication adherence can change with time, the same can be said about the factors that influence it. Recent studies implementing a risk model based on multinomial logistic regressions do not allow this type of characterization [38,39,40,42]. This is because the traditional approach is limited to reporting adjusted odds ratios, representing the association between predictors and trajectory memberships, all else equal [48,49].

Time-fixed models found several risk factors associated with non-adherence, including predisposing characteristics such as being female, foreign-born, or non-white. These results align with previous studies linking non-adherence to demographic factors [7,23,50,51,52,53]. Even though college education was not found to be a significant risk factor for belonging to at least one non-adherent trajectory in any of the three models, a similar study linking administrative health care claims to a population-level survey from Australia reported similar findings when education was adjusted for covariates similar to ones considered in this study [50].

The multitrajectory model revealed that enabling characteristics like self-reported health, depression symptoms, and life satisfaction significantly predicted non-adherence. While time-fixed models linked non-adherence to depression, smoking, and drinking, the multitrajectory analysis showed stable probabilities for smoking and drinking but highlighted dynamic shifts in Medicaid eligibility, additional health coverage, and independence levels (IADL/ADL). Notably, additional health coverage, non-significant in time-fixed models, was strongly linked to high adherence in the multitrajectory analysis. It is important to clarify that variations in these characteristics do not imply a causal relationship but rather a longitudinal description of how each adherence trajectory and covariate trajectory progressed with time.

In essence, the time-fixed approach exhibited inconsistency in identifying which predictors were statistically significant factors of each medication adherence trajectory across pharmacotherapy classes. If researchers use only a time-fixed approach, results can exhibit statistical significance or not, like in this study. In case of non-statistical significance, the strength of evidence to guide actual practice innovations could be hampered. However, using the time-varying approach, researchers can look at the trajectory of individual predictors and determine if there is an actual variation over time that could be clinically meaningful. Practitioners can then investigate whether those predictor variations over time are worth tackling in practice to improve medication adherence.

This study emphasizes the value of multitrajectory modeling in identifying predictors of non-adherence linked to significant changes over time. This approach helps health care providers pinpoint key aspects of a patient’s life requiring intervention, such as the loss of a caregiving spouse, secondary health coverage, or autonomy. Health care providers and pharmacists could proactively assess the patient’s circumstance and identify causes of non-adherence (e.g., frailty, changes in household support). This would require building data-reporting systems that include contextual information about what the patient is going through that could be identified as a risk for non-adherence. Additionally, health care systems could implement data systems that allow for longitudinal measurements of medication adherence for individual patients, instead of measuring the PDC dichotomously during predetermined periods (i.e., the annual PDC). By characterizing these predictors throughout time, a multitrajectory analysis can guide targeted interventions and referrals, tailoring care to the specific needs of the patient population.

### Limitations

Several limitations exist. Risk factors were drawn from the HRS using the ABM framework, but the HRS was not specifically designed to measure medication adherence predictors. Firstly, this study used an initial model of medication adherence trajectories that were estimated from administrative health care claims. While this approach for measuring adherence has been validated extensively, including by Grymonpre et al. and Galozy and colleagues, there are recognizable pitfalls associated with using these data [54,55]. These include patients filling prescriptions without insurance, via cash purchases or using promotional coupons (e.g., GoodRx or equivalent) that might not be recorded as claims and therefore indicate non-adherence, when, in reality, the patient had medication in hand. Despite the potential pitfalls of the PDC as a proxy measure for medication adherence, PDC estimates from administrative data sources have shown to correlate well with other direct observation methods to inform medication adherence [56]. High rates of missing data may have affected significance in time-fixed models and biased multitrajectory analysis. Additionally, there was a mismatch in measurement periods—adherence was estimated monthly using the PDC, while risk factors were measured biennially in the HRS. Despite these limitations, this study highlights the multitrajectory analysis as a promising method for exploring the impact of time-varying predictors on adherence. Moreover, this study did not examine the impact of adverse events in the trajectories of medication adherence, such as myocardial infarction or stroke. Such events have been previously described as predictors of poor medication adherence. Future studies following a quasi-experimental approach could explore the impact of acute negative events in the trajectories of medication adherence to chronic medications. Finally, this study included data obtained from HRS, which were obtained via surveys, which could be subject to potential recall bias.

## 5. Conclusions

This study demonstrated the potential of multitrajectory modeling to identify time-varying risk factors for non-adherence. Unlike traditional multinomial regression, this approach identifies both static and dynamic predictors, offering insights into which factors meaningfully change over time. Such methods can guide targeted interventions, improve medication adherence, and better support at-risk patient populations.

## Figures and Tables

**Table 1:** Operationalization of the dimensions of two conceptual frameworks: the Andersen’s Behavioral Model of Health Services Use and the causes of non-adherence summarized by the World Health Organization.

		WHO Report: Causes of Non-Adherence				
		Socioeconomic	Health care team/Health care system	Disease-related factors	Therapy-related factors	Patient-related factors
**Andersen’s Behavioral Model of Health Services Use**	Predisposing characteristics	Education, race *, ethnicity *, income *, occupation, marital status *	Trust in medical organizations/health care team	Health beliefs		Transportation, distance to health services, substance abuse *
	Enabling factors	Urbanicity, Medicaid eligibility *	Access to health care services, wait times, difficulty filling prescriptions, cost, health information, integration of health care team, physician–patient communication, Facetime with health care providers			Health insurance *, social/family support *, health literacy
	Need characteristics			Evaluated health status *, comorbidities * (MI, stroke, cancer), severity, symptoms *	Treatment complexity, route of administration, side effects, duration, degree of behavioral change required	Activities of daily living *, limitations in activities/profession *, risk factors (obesity, smoking, alcohol use) *

* Concept identified in the HRS public survey and included in the analysis.

**Table 2:** Study sample of sociodemographic, enabling, and need characteristics.

Sample Characteristics	Frequency of Study Participants (n,%)	Missing Data
N = 11,068		(n, %)
Predisposing and antecedents		
Sex (n = 11,068)		0, 0%
*Female*	6724, 60.75%	
Birthplace (n = 9564)		1504, 13.58%
*US-born*	8475, 88.61%	
Race (n = 11,057)		11, 0.09%
*Non-white*	2597, 23.49%	
Ethnicity (n = 11,058)		10, 0.09%
*Hispanic*	1302, 11.77%	
Education (n = 11,068)		0, 0%
Has college degree or higher	2263, 20.45%	
Enabling characteristics		
Self-reported health status (n = 6308)		4760, 43.01%
*Excellent*	282, 4.47%	
*Very good*	1349, 21.39%	
*Good*	2127, 33.72%	
*Fair*	1826, 28.95%	
*Poor*	724, 11.48%	
Depression symptoms (n = 9432)		1636, 14.78%
With clinical depression *	1919, 20.35%	
Life satisfaction (n = 1761)		9307, 84.09%
*Completely satisfied*	395, 22.43%	
*Very satisfied*	726, 41.23%	
*Somewhat satisfied*	528, 29.98%	
*Not very satisfied*	85, 4.83%	
*Not at all satisfied*	27, 1.53%	
Retirement satisfaction (n = 4667)		6401, 57.83%
*Very Satisfied*	2132, 45.68%	
*Moderately satisfied*	2048, 43.88%	
*Not at all satisfied*	487, 10.43%	
Limitations in work due to health (n = 5977)		5091, 46.00%
*Yes*	3435, 57.47%	
Need characteristics		
Poverty index (n = 9609)		1459, 13.18%
*Household income below poverty threshold*	1426, 14,84%	
Marital status (n = 9805)		1263, 11.41%
Loss of spouse or never married **	5404, 55.11%	
*Lives with spouse, partner*	4401, 44.89%	
Number of resident children (n = 6320)		4748, 42,90%
*Does not live with resident children*	4852, 76.77%	
*Lives with resident children*	1468, 23.23%	
Medicaid eligibility (n = 9798)		1270, 11.47%
*Medicaid beneficiary*	2007, 20.48%	
Additional health insurance coverage (n = 6216)		4852, 43,84%
*Has additional insurance*	1920, 30.89%	
Smoking status (n = 9749)		1319, 11.91%
*Smokers*	986, 10.11%	
Number of drinking days per week (n = 6294)		4774, 43.13%
*0 or does not* *drink*	4473, 71.07%	
*1*	658, 10.45%	
*2*	304, 4.83%	
*3*	245, 3.89%	
*4*	102, 1.62%	
*5*	124, 1.97%	
*6*	52, 0.83%	
*7*	336, 5.34%	
Instrumental activities of daily living (n = 9822)		1246, 11.25%
*0 (Highly functional)*	7458, 75.93%	
*1*	1035, 10.54%	
*2*	605, 6.16%	
*3 (Not functional)*	724, 7.37%	
Activities of daily living (n = 9822)		1246, 11.25%
*0 (Completely independent)*	6316, 64.3%	
*1*	1160, 11.81%	
*2*	735, 7.48%	
*3*	504, 5.13%	
*4*	486, 4.95%	
*5 (Totally dependent)*	621, 6.32%	
Pharmacotherapeutic class ***		
*Select antihypertensives*	7727, 69.81%	
*Blood cholesterol lowering drugs*	8221, 74.28%	
*Oral diabetes medications*	3214, 29.04%	

* The CESD-8 (Center for Epidemiologic Studies Depression 8-item) scale is a validated instrument to measure depressive symptoms. Per Steffick and colleagues, a score > 3 is indicative of clinical depression [24]. ** Loss of spouse due to death, separation, or divorce. *** Participants could be taking a concomitant drug from more than one pharmacotherapeutic class.

**Table 3:** Time-fixed predictors of the rapid decline trajectory of the select antihypertensives, statins, and diabetes medications in medication adherence trajectory models.

TRAJECTORY	Rapid Decline ^a^											
GBTM MODEL	Select Antihypertensives				Statins				Oral Diabetes Medications			
	Coeff.	S.E.	aOR	*p*-Value	Coeff.	S.E.	aOR	*p*-Value	Coeff.	S.E.	aOR	*p*-Value
Predisposing and antecedents												
Sex: Female	0.11	0.12	1.11	0.392	0.16	0.15	1.18	0.273	−0.01	0.29	0.99	0.980
Birthplace: Foreign-born	0.00	0.21	1.00	0.988	0.91	0.24	2.48	0.000 *	0.19	0.44	1.21	0.673
Race: Non-white	−0.01	0.14	0.99	0.938	0.16	0.18	1.18	0.374	0.15	0.30	1.16	0.630
Ethnicity: Hispanic	−0.25	0.22	0.78	0.247	−0.13	0.26	0.88	0.619	0.08	0.42	1.08	0.848
Education: Not college-educated	−0.03	0.18	0.97	0.858	0.52	0.22	1.67	0.018 *	0.21	0.40	1.23	0.606
Enabling characteristics												
Self-reported Health Status	0.03	0.07	1.03	0.646	0.00	0.08	1.00	0.98	0.10	0.17	1.11	0.540
Depression Symptoms	0.60	0.17	1.82	0.000 *	0.39	0.22	1.48	0.07	0.16	0.41	1.18	0.691
Life Satisfaction	0.16	0.07	1.17	0.025 *	0.02	0.09	1.02	0.86	0.14	0.16	1.15	0.392
Retirement Satisfaction	−0.03	0.10	0.97	0.753	0.09	0.12	1.10	0.45	0.17	0.22	1.18	0.455
Limitations in Work Due to Health	0.17	0.13	1.19	0.181	0.22	0.16	1.25	0.16	0.31	0.30	1.37	0.306
Need characteristics												
Household income below poverty index	0.12	0.18	1.13	0.512	0.00	0.24	1.00	1.00	0.13	0.42	1.14	0.756
Marital status: Loss of spouse	0.01	0.02	1.01	0.617	−0.01	0.03	0.99	0.81	0.06	0.05	1.06	0.293
Lives with resident children	0.03	0.11	1.03	0.769	0.09	0.14	1.10	0.51	−0.16	0.25	0.85	0.509
Medicaid beneficiary	−0.11	0.17	0.90	0.539	0.13	0.22	1.14	0.55	−1.39	0.54	0.25	0.010 *
Additional health coverage	−0.02	0.13	0.98	0.869	0.01	0.15	1.01	0.95	0.30	0.30	1.35	0.307
Smoking status: Smoker	0.40	0.18	1.49	0.028 *	0.76	0.22	2.13	0.00 *	0.85	0.43	2.35	0.046 *
Number of drinking days/week	0.04	0.03	1.04	0.159	−0.02	0.04	0.98	0.58	−0.11	0.10	0.89	0.263
Instrumental activities of daily living	0.01	0.12	1.01	0.937	0.63	0.15	1.88	0.00 *	−0.09	0.31	0.91	0.768
Activities of daily living	0.09	0.06	1.09	0.158	−0.14	0.08	0.87	0.10	−0.01	0.16	0.99	0.937

^a^ The trajectory of rapid decline in medication adherence is observed for all of the models of select antihypertensives, statins, and diabetes medications. * Statistically significant at the 0.05 level.

**Table 4:** Time-fixed predictors of the slow decline trajectory of the select antihypertensives, statins, and diabetes medications in medication adherence trajectory models.

TRAJECTORY	Slow Decline ^a^											
GBTM MODEL	Select Antihypertensives				Statins				Oral Diabetes Medications			
	Coeff.	S.E.	aOR	*p*-Value	Coeff.	S.E.	aOR	*p*-Value	Coeff.	S.E.	aOR	*p*-Value
Predisposing and antecedents												
Sex: Female	0.10	0.09	1.11	0.254	−0.02	0.11	0.98	0.846	0.21	0.18	1.24	0.245
Birthplace: Foreign-born	0.03	0.14	1.03	0.831	0.10	0.20	1.10	0.637	0.66	0.28	1.93	0.017 *
Race: Non-white	0.37	0.10	1.44	0.000 *	0.23	0.13	1.26	0.084	−0.09	0.20	0.91	0.645
Ethnicity: Hispanic	0.04	0.14	1.04	0.784	0.15	0.19	1.17	0.421	−0.56	0.28	0.57	0.050 *
Education: Not college-educated	0.06	0.13	1.06	0.633	0.24	0.16	1.27	0.143	0.34	0.27	1.40	0.212
Enabling characteristics												
Self-reported Health Status	0.22	0.05	1.24	0.000 *	0.15	0.06	1.16	0.013 *	0.14	0.10	1.14	0.188
Depression Symptoms	0.23	0.13	1.26	0.066	0.21	0.16	1.23	0.198	0.50	0.25	1.65	0.042 *
Life Satisfaction	−0.04	0.05	0.96	0.398	0.02	0.06	1.02	0.817	0.05	0.10	1.05	0.654
Retirement Satisfaction	−0.04	0.07	0.96	0.588	0.09	0.09	1.09	0.325	−0.09	0.14	0.91	0.510
Limitations in Work Due to Health	0.04	0.09	1.04	0.700	0.17	0.11	1.19	0.133	0.35	0.19	1.42	0.065
Need characteristics												
Household income below poverty index	0.05	0.13	1.05	0.697	0.31	0.18	1.37	0.075	−0.30	0.26	0.74	0.259
Marital status: Loss of spouse	0.01	0.02	1.01	0.562	−0.01	0.02	0.99	0.506	0.01	0.03	1.01	0.693
Lives with resident children	0.09	0.08	1.09	0.254	0.15	0.10	1.16	0.145	0.13	0.13	1.14	0.322
Medicaid beneficiary	−0.11	0.12	0.90	0.384	0.02	0.17	1.02	0.931	0.01	0.24	1.01	0.982
Additional health coverage	−0.18	0.09	0.84	0.057	0.17	0.11	1.19	0.112	0.46	0.19	1.59	0.016 *
Smoking status: Smoker	−0.03	0.15	0.97	0.855	0.11	0.19	1.12	0.543	0.09	0.32	1.09	0.784
Number of drinking days/week	0.05	0.02	1.06	0.017 *	0.00	0.03	1.00	0.986	0.04	0.05	1.04	0.459
Instrumental activities of daily living	0.05	0.09	1.06	0.557	0.42	0.12	1.52	0.000	0.27	0.17	1.31	0.104
Activities of daily living	0.01	0.05	1.01	0.783	−0.09	0.06	0.92	0.151	−0.01	0.09	0.99	0.949

^a^ The trajectory of slow decline in medication adherence is observed for all of the models of select antihypertensives, statins, and diabetes medications. * Statistically significant at the 0.05 level.

**Table 5:** Time-fixed predictors of the moderate decline trajectory of the select antihypertensives, statins, and diabetes medications in medication adherence trajectory models.

TRAJECTORY	Moderate Decline ^a^											
GBTM MODEL	Select Antihypertensives				Statins				Oral Diabetes Medications			
	Coeff.	S.E.	aOR	*p*-Value	Coeff.	S.E.	aOR	*p*-Value	Coeff.	S.E.	aOR	*p*-Value
Predisposing and antecedents												
Sex: Female	-	-	-	-	0.40	0.12	1.50	0.001 *	0.25	0.17	1.28	0.149
Birthplace: Foreign-born	-	-	-	-	0.56	0.20	1.75	0.006 *	0.05	0.27	1.05	0.862
Race: Non-white	-	-	-	-	0.69	0.14	2.00	0.000 *	−0.37	0.19	0.69	0.054
Ethnicity: Hispanic	-	-	-	-	0.20	0.20	1.23	0.311	0.14	0.25	1.15	0.564
Education: Not college-educated	-	-	-	-	0.07	0.18	1.07	0.704	0.25	0.26	1.28	0.333
Enabling characteristics												
Self-reported Health Status	-	-	-	-	0.09	0.07	1.09	0.196	0.14	0.10	1.15	0.153
Depression Symptoms	-	-	-	-	0.23	0.17	1.26	0.175	0.79	0.23	2.20	0.001 *
Life Satisfaction	-	-	-	-	0.10	0.07	1.10	0.159	0.13	0.10	1.14	0.187
Retirement Satisfaction	-	-	-	-	0.14	0.10	1.15	0.137	−0.01	0.14	0.99	0.966
Limitations in Work Due to Health	-	-	-	-	0.02	0.13	1.02	0.889	0.08	0.18	1.08	0.677
Need characteristics												
Household income below poverty index	-	-	-	-	0.25	0.18	1.29	0.163	−0.30	0.25	0.74	0.231
Marital status: Loss of spouse	-	-	-	-	−0.02	0.02	0.98	0.326	0.00	0.03	1.00	0.973
Lives with resident children	-	-	-	-	0.02	0.11	1.02	0.836	0.00	0.13	1.00	0.997
Medicaid beneficiary	-	-	-	-	0.21	0.17	1.23	0.223	−0.09	0.23	0.92	0.706
Additional health coverage	-	-	-	-	−0.08	0.13	0.92	0.529	0.01	0.19	1.01	0.978
Smoking status: Smoker	-	-	-	-	0.38	0.19	1.46	0.049 *	0.18	0.31	1.19	0.566
Number of drinking days/week	-	-	-	-	−0.05	0.03	0.96	0.165	−0.08	0.06	0.92	0.149
Instrumental activities of daily living	-	-	-	-	0.24	0.13	1.27	0.061	−0.07	0.17	0.93	0.684
Activities of daily living	-	-	-	-	−0.09	0.07	0.92	0.179	0.01	0.09	1.01	0.871

^a^ The trajectory of moderate decline in medication adherence is observed only in the models of statins and diabetes medications. * Statistically significant at the 0.05 level.

**Table 6:** Time-fixed predictors of the low then increasing adherence trajectory of the statins, and diabetes medications in medication adherence trajectory models.

TRAJECTORY	Low then Increasing Adherence ^a^											
GBTM MODEL	Select Antihypertensives				Statins				Oral Diabetes Medications			
	Estimate	S.E.	aOR	*p*-Value	Estimate	S.E.	aOR	*p*-Value	Estimate	S.E.	aOR	*p*-Value
Predisposing and antecedents												
Sex: Female	-	-	-	-	0.06	0.10	1.06	0.561	0.71	0.20	2.02	0.001 *
Birthplace: Foreign-born	-	-	-	-	0.48	0.18	1.62	0.009 *	0.05	0.29	1.05	0.868
Race: Non-white	-	-	-	-	0.30	0.13	1.35	0.019 *	0.26	0.20	1.30	0.189
Ethnicity: Hispanic	-	-	-	-	0.02	0.18	1.02	0.930	0.14	0.27	1.15	0.599
Education: Not college-educated	-	-	-	-	0.15	0.15	1.16	0.334	−0.47	0.32	0.63	0.136
Enabling characteristics												
Self-reported Health Status	-	-	-	-	0.08	0.06	1.08	0.168	0.01	0.11	1.01	0.946
Depression Symptoms	-	-	-	-	0.19	0.15	1.21	0.213	0.71	0.26	2.04	0.005 *
Life Satisfaction	-	-	-	-	−0.11	0.06	0.90	0.078	0.32	0.11	1.37	0.004 *
Retirement Satisfaction	-	-	-	-	0.16	0.08	1.17	0.055	0.02	0.15	1.02	0.897
Limitations in Work Due to Health	-	-	-	-	0.17	0.11	1.18	0.114	0.15	0.21	1.17	0.456
Need characteristics												
Household income below poverty index	-	-	-	-	−0.12	0.17	0.88	0.473	0.00	0.26	1.00	0.990
Marital status: Loss of spouse	-	-	-	-	−0.03	0.02	0.97	0.098	−0.03	0.04	0.97	0.420
Lives with resident children	-	-	-	-	0.07	0.10	1.07	0.467	−0.24	0.16	0.79	0.121
Medicaid beneficiary	-	-	-	-	0.16	0.15	1.17	0.310	−0.34	0.25	0.71	0.174
Additional health coverage	-	-	-	-	−0.06	0.10	0.94	0.574	−0.32	0.23	0.73	0.168
Smoking status: Smoker	-	-	-	-	0.16	0.18	1.17	0.368	0.46	0.32	1.58	0.150
Number of drinking days/week	-	-	-	-	−0.02	0.03	0.98	0.454	0.01	0.06	1.01	0.885
Instrumental activities of daily living	-	-	-	-	0.08	0.12	1.09	0.490	−0.07	0.18	0.93	0.681
Activities of daily living	-	-	-	-	0.01	0.06	1.01	0.843	0.10	0.09	1.10	0.283

^a^ The trajectory of low then increasing medication adherence is observed only in the models of statins and diabetes medications. * Statistically significant at the 0.05 level.

**Table 7:** Time-fixed predictors of the high then increasing adherence trajectory of the diabetes medications in medication adherence trajectory models.

TRAJECTORY	High then Increasing Adherence ^a^											
GBTM MODEL	Select Antihypertensives				Statins				Oral Diabetes Medications			
	Estimate	S.E.	aOR	*p*-Value	Estimate	S.E.	aOR	*p*-Value	Estimate	S.E.	aOR	*p*-Value
Predisposing and antecedents												
Sex: Female	-	-	-	-	-	-	-	-	0.23	0.19	1.26	0.221
Birthplace: Foreign-born	-	-	-	-	-	-	-	-	0.20	0.30	1.22	0.511
Race: Non-white	-	-	-	-	-	-	-	-	−0.36	0.21	0.70	0.092
Ethnicity: Hispanic	-	-	-	-	-	-	-	-	−0.43	0.30	0.65	0.146
Education: Not college-educated	-	-	-	-	-	-	-	-	−0.31	0.30	0.74	0.313
Enabling characteristics												
Self-reported Health Status	-	-	-	-	-	-	-	-	0.08	0.10	1.08	0.444
Depression Symptoms	-	-	-	-	-	-	-	-	0.29	0.25	1.34	0.253
Life Satisfaction	-	-	-	-	-	-	-	-	0.01	0.11	1.01	0.947
Retirement Satisfaction	-	-	-	-	-	-	-	-	−0.19	0.15	0.83	0.212
Limitations in Work Due to Health	-	-	-	-	-	-	-	-	0.11	0.20	1.12	0.571
Need characteristics												
Household income below poverty index	-	-	-	-	-	-	-	-	−0.40	0.27	0.67	0.147
Marital status: Loss of spouse	-	-	-	-	-	-	-	-	0.03	0.04	1.03	0.465
Lives with resident children	-	-	-	-	-	-	-	-	−0.09	0.15	0.91	0.527
Medicaid beneficiary	-	-	-	-	-	-	-	-	0.08	0.25	1.08	0.747
Additional health coverage	-	-	-	-	-	-	-	-	0.09	0.21	1.09	0.686
Smoking status: Smoker	-	-	-	-	-	-	-	-	0.22	0.33	1.25	0.504
Number of drinking days/week	-	-	-	-	-	-	-	-	−0.06	0.06	0.94	0.342
Instrumental activities of daily living	-	-	-	-	-	-	-	-	0.05	0.18	1.05	0.770
Activities of daily living	-	-	-	-	-	-	-	-	0.12	0.09	1.12	0.201

^a^ The trajectory of high then increasing medication adherence is observed only in the models of diabetes medications. * Statistically significant at the 0.05 level.

## Data Availability

Data pertaining to the medication adherence trajectories is considered restricted data and therefore cannot be made available publicly, according to the Data Use Agreement with National Institute of Aging. Nevertheless, the data pertaining to the predictors of medication adherence trajectories utilized in this study was obtained from the public survey of the Health and Retirement Study, available at https://hrsdata.isr.umich.edu/data-products/public-survey-data.

## Abbreviations

The following abbreviations are used in this manuscript:

GBTM	Group-based trajectory modeling
PDC	Proportion of days covered
HRS	Health and Retirement Study
VIF	Variance inflation factor
ADLs	Activities of Daily Living
IADLs	Instrumental Activities of Daily Living

## Appendix A

**Table A1:** Operationalization of enabling and need characteristics covariates.

Characteristic	Covariates	Measurement Approach
Enabling characteristics	Self-reported health status	5-point scale: 1—Excellent 2—Very good 3—Good 4—Fair 5—Poor
	Depression symptoms	CES-D 8-Item Scale. Per Steffick and colleagues, a score > 3 is indicative of clinical depression [24] 0—No depression symptoms (CES-D score ≤ 3) 1—With depression symptoms (CES-D score > 3)
	Life satisfaction	5-point scale: 1—Completely satisfied 2—Very satisfied 3—Somewhat satisfied 4—Not very satisfied 5—Not at all satisfied
	Retirement satisfaction	3-point scale: 1—Very satisfying 2—Moderately satisfying 3—Not at all satisfying
	Limitations in work due to health	Yes (1)/No (0)
Need characteristics	Poverty threshold	Below (1)/Above (0)
	Family structure	
	Loss of spouse	Yes (1)/No (0)
	Living with resident children	Yes (1)/No (0)
	Medicaid beneficiary	Yes (1)/No (0)
	Additional health coverage	Yes (1)/No (0)
	Substance abuse	
	Smoking status	Yes (1)/No (0)
	Alcohol consumption	Number of drinking days/week
	Assistance with activities Instrumental activities of daily living (IADLs) Activities of daily living (ADLs)	Number of activities requiring assistance/cannot perform

CES-D: The 8-item Center for Epidemiological Studies Depression Scale [57].

## Appendix B

Given the possibility of multicollinearity, the variance inflation factor (VIF) was computed to determine by how much each risk factor estimate is increased because of high correlation with other risk factors. VIF and R^2^ were computed to examine the presence of multicollinearity for the risk factors in each adjusted group-based trajectory model. In general, a VIF greater than 5 is indicative of multicollinearity.

**Table A2:** Multicollinearity Analysis.

GBTM Model	Select Hypertensives		Statins		Diabetes	
Covariate	VIF	R^2^	VIF	R^2^	VIF	R^2^
Predisposing and antecedents						
Sex: Female	1.170	0.144	1.150	0.130	1.220	0.179
Birthplace: Foreign-born	1.430	0.299	1.470	0.320	1.590	0.372
Race: Non-white	1.200	0.165	1.180	0.153	1.190	0.162
Ethnicity: Hispanic	1.530	0.347	1.550	0.357	1.740	0.425
Education: Not college-educated	1.820	0.451	1.850	0.459	1.790	0.440
Enabling characteristics						
Self-reported Health Status	1.550	0.355	1.500	0.332	1.470	0.319
Depression Symptoms	1.930	0.482	2.010	0.502	1.960	0.490
Life Satisfaction	1.280	0.216	1.260	0.204	1.260	0.205
Retirement Satisfaction	1.310	0.237	1.320	0.242	1.240	0.191
Limitations in Work Due to Health	1.270	0.211	1.290	0.224	1.300	0.232
Need characteristics						
Household income below poverty index	1.340	0.252	1.330	0.251	1.340	0.256
Marital spouse: Loss of spouse	1.220	0.182	1.200	0.169	1.280	0.218
Number of resident children	1.080	0.074	1.080	0.075	1.060	0.057
Medicaid eligibility	1.320	0.245	1.320	0.241	1.360	0.263
Additional health coverage	1.130	0.118	1.120	0.110	1.180	0.155
Smoking status: Smoker	1.050	0.051	1.060	0.054	1.030	0.029
Number of drinking days/week	1.140	0.119	1.100	0.093	1.080	0.070
Instrumental activities of daily living	1.360	0.263	1.410	0.289	1.550	0.356
Activities of daily living	1.510	0.336	1.510	0.339	1.760	0.432
Mean VIF	1.381		1.389		1.410	
